# Aqueous dispersions of carbon black and its hybrid with carbon nanofibers

**DOI:** 10.1039/c8ra05446k

**Published:** 2018-09-17

**Authors:** Mohamed Youssry, Fadi Z. Kamand, Musaab I. Magzoub, Mustafa S. Nasser

**Affiliations:** Department of Chemistry and Earth Sciences, College of Arts and Sciences, Qatar University P. O. Box 2713 Doha Qatar myoussry@qu.edu.qa +974-44036541; Gas Processing Center, College of Engineering, Qatar University P. O. Box 2713 Doha Qatar

## Abstract

The aqueous dispersions of a special type of carbon black (CB) in 1 M lithium bis(trifluoromethanesulfonimide) electrolyte is mainly controlled by the affinity of the aqueous electrolyte towards the CB particles rather than the particle size. In spite of its small particle size (30 nm), this type of CB forms a three-dimensional open network which is rheologically and electrically percolated at a relatively high threshold (2.0 wt%) with enhanced rheological and electrical properties. At this percolating threshold, replacing a trace amount of CB with equivalent carbon nanofibers (CNFs) produces hybrid dispersions with higher electrical conductivity and comparable rheological behavior to pure CB dispersions. This hybrid dispersion is dominated by a cooperatively supporting network, which is wired by the flexible filamentous nanofibers so that it is able to recover the conductivity loss under flow conditions due to flow-induced breaking up of the conductive pathways of CB and presumably sustain a higher load of active materials. This finding suggests hybrid dispersions as a promising precursor in the formulation of electrode suspensions for aqueous semi-solid redox flow cells.

## Abstract 2

Optimal hybrid dispersion of carbon black (CB) and nanofibers (CNFs) is formed at a critical content of CNFs before its aggregation concentration so that CNFs wire CB aggregates to recover the conductivity loss without increasing of CB rigidity.

## Graphical abstract

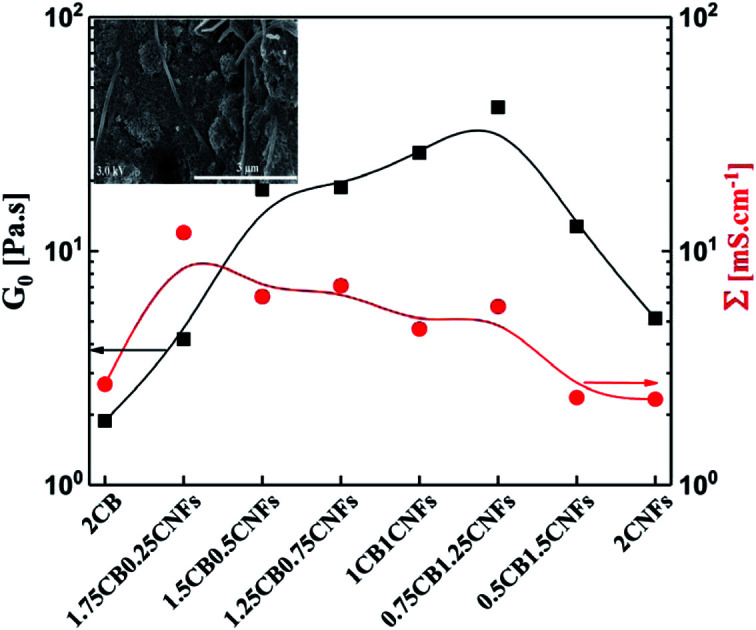

## 1. Introduction

Carbon black (CB) is an important member of a large family of carbon nanomaterials that are allotropes of carbon and characterized by their exceptional chemical stability; electrical and mechanical properties derive from their large diversity of structures that extend from the sp^2^-hybridization of carbon atoms to the crystalline structure (varied degree of graphitization). Among various kinds of carbon nanomaterials, filamentous (one-dimensional) carbon nanofibers (CNFs) and carbon nanotubes (CNTs), platelet-like (two-dimensional) graphite and graphene, and most commonly semi-spherical (three-dimensional) carbon black (CB) have a unique place in many applications including printing inks, paints and importantly the formulations of electrodes for energy storage systems (batteries and supercapacitors).^1,2^

Carbon black is initially formed from semi-spherical primary particles of nanometric (few hundred nm) size, which are fused to form aggregates (few μm size scale) in dispersions.^3^ The van der Waals attraction forces drive the aggregates to binding into larger loosely agglomerates (10–100 μm).^4^ The specific surface area and surface chemistry of CBs play pivotal role in determine the strength of binding forces^5^ which determines the microstructure and hence the flow behavior of CB suspensions.^6^ Above a critical concentration, the so-called percolating threshold, a three-dimensional network is formed across the CB dispersion showing abrupt increase of the rheological parameters (viscosity, shear modulus) and electrical conductivity of dispersion.^4,7^ This behavior is a consequence of formation of percolated network composed of branches (paths) of aggregated CB resulting in 3D network.^4^ It has been reported that the particle size of CBs impacts on the their rheological behavior where large particles (with relatively small specific surface area) form weaker percolated network consisting of less dense agglomerates with numerous branches and hence lower rheological parameters and electrical conductivity.^4^ Of course, the surface chemistry of CBs, dispersing medium and dissolved salt (type and concentration) might influence the rheological and electrical behavior of CB suspensions.^4^

Recently, Duduta *et al.*^8^ introduced a novel approach for energy storage system; the so-called semi-solid redox flow cell (SSFC) in which the electrode suspension is typically composed of electroactive materials and electronic conductive nanoparticles suspended in nonaqueous medium. Thereafter, the focus of research was devoted to nonaqueous-based cells^9–11^ until the pioneer study by Li *et al.*^12^ which highlighted the importance of replacing the nonaqueous cells by alternative aqueous-based cells, offering scalable low-cost storage. A fundamental requirement for these cells is the formation of percolated network of electronically conductive CB particles. This network should sufficiently sustain as high amount of electroactive (electrically insulating) materials as possible for enhanced energy capacity but still has considerable flowability. Youssry *et al.* reported on the rheological and electrical properties of two kinds of carbon blacks, namely ketjen black (KB) and super C45, in organic medium.^4,13^ It has been shown that the particle size (surface area), surface chemistry as well as the affinity of dispersing medium towards the CB particles play crucial role in determining the microstructure and percolating threshold: smaller the particle size, lower the percolating threshold is.^4^ However, incorporation of relatively large electroactive materials negatively influence the percolated network of carbon black by rupturing the conductive branches resulting in serious losing of electrical connection.^13^ Moreover, the CB dispersions under flow conditions suffer shear-induced structural transitions when the network breaks up into small aggregates leading to strong drop in the electrical conductivity.^4^

The filamentous morphology (high aspect ratio > 100), high electrical conductivity, mechanical strength, and chemical and thermal stability of CNFs renders them a focus of recent research studies to be conductive additives alternative to CBs in the formulation of electrodes for energy storage systems.^13–15^ However, several challenges still impede the implementation of CNFs in the formulation of electrodes for commercial applications. This is due to the CNF-based electrodes have critical drawbacks arise from the high irreversible capacity and short life cycle^16^ as well as the dependency of the average diameter and length of CNFs on the preparation method. However, their low cost, relatively larger diameters and lengths (and hence weaker van der Waals forces) render CNFs more feasible to be conductive additives in the formulation of electrodes in the form of hybrid with CBs.

To overcome the conductivity loss of CBs, either due to incorporation of electroactive materials or shear-induced breaking up of CB branches, Youssry *et al.* suggested a novel approach of hybrid dispersion where trace amount of carbon nanofibers (CNFs) replaced an equivalent amount of CB at its percolating threshold.^17^ The CB-CNFs hybrid dispersions acquire the advantages of both carbon nanomaterials and their percolated network could sustain higher loading of electrically-insulating electroactive materials and recover the flow-induced conductivity loss *via* wiring CB branches by the flexible nanofibers.^17^ Similarly, Cho *et al.*^18^ showed that hybrid conductive additives of carbon Super-P and vapor-grown carbon fibers had a synergistic effect on the electrochemical performance of different cathode materials for lithium-ion batteries.

To our knowledge, no systematic study (except few work^19^) has been appeared so far addressing the influence of the characteristic parameters on the rheological and electrical properties of CB dispersions in aqueous media. Such study is mandatory since CBs are the conductive additives used in commercial energy storage systems such as lithium-ion batteries (at concentration of *ca.* 2 wt%) regardless the characteristics of CB and wettability of dispersing medium. Moreover, there is a direction towards minimizing the utilization of organic solvents and electrolytes and alternatively use aqueous medium in electrode formulation due to economic and environmental impacts.

In line with the novel trend towards utilization of aqueous electrode suspensions, this work aims to: (i) systematically study the evolution of microstructure and shear-induced structural transitions in dispersions of commercial carbon black (PBX51) in aqueous electrolyte (1 M lithium bis(trifluoromethanesulfonimide) in water) at ambient temperature (25 °C), (ii) comparatively account for the effect of dispersing medium (aqueous *versus* nonaqueous electrolyte) on the percolating threshold, and (iii) formulate aqueous hybrid dispersions of carbon black and nanofibers with optimal rheological and electrical properties as a precursor conductive additive for aqueous semi-solid redox flow cells.

## 2. Experimental

### 2.1. Materials and preparation of aqueous dispersions

Carbon black (CB) and lithium bis(trifluromethanesulphonyl)imide (LiTFSI) were gifts from Cabot Corporation, USA and 3M, France, respectively. The carbon black has a manufacturer code: PBX51 with specific surface area of 1420 m^2^ g^−1^. The carbon nanofibers (CNFs) were purchased from Sigma Aldrich, USA, having a length of 20–200 μm and an average dimeter of 100 nm, with specific surface area of 24 m^2^ g^−1^. All materials were used as received without any treatment. Deionized water was the solvent in this study.

The dispersions were prepared using planetary ball mill (Changsha Tianchuang Powder Technology Co., Ltd XQM-0.4A) equipped by agate grinding jars with three agate balls (diameter 6 mm). Weighted amounts of carbon materials were dispersed in 1 M LiTFSI aqueous solution for 3 hours at 500 rpm at room temperature (*ca.* 23 °C).

### 2.2. Rheological measurements

The rheological behaviour of dispersion was measured using a stress-controlled rotational rheometer (Anton Paar MCR-302, Austria). Two geometries were used depending on the sample fluidity: steel plate–plate (plate diameter 50 mm, 1 mm gap size) geometry for high-viscosity dispersions and concentric cylinder for low-viscosity ones. To remove the shear history, the dispersions were pre-sheared at shear rate of 500 s^−1^ for 5 min, and then left at rest for 20 min before starting the rheological measurement. In order to determine the linear viscoelastic region, the strain sweep test was conducted between over strain (*γ*) range of 0.01–100%, and then the sample remained at rest for 10 min before recording the dynamic frequency sweep experiment. The frequency sweep tests were conducted over angular frequency range of 0.1–1000 rad.s^−1^ at adequate strain amplitude (*γ*_0_) in the linear viscoelastic region which guarantee the minimal perturbation for the microstructure of the dispersions. The steady rheology expressed by the flow curve (viscosity (*η*) with shear rate (*γ̇*)) was recorded over shear range of 0.01–1000 s^−1^. All rheological measurements were conducted at 25 °C controlled by Peltier system.

### 2.3. Zeta potential measurements

The zeta potential (*ζ*) was measured by Zetasizer Nanoseries (Malvern Instruments™ Ltd). Highly diluted dispersions of CB in varied concentrations of LiTFSI solution were prepared by mixing a drop of stock CB dispersion in given concentration of LiTFSI. In bath sonicator, the samples were homogenized for 20–30 min before the measurements, which had been conducted at 25 °C.

### 2.4. Impedance spectroscopy

The electrochemical impedance spectra were collected by using CorrTest CS350 potentiostat/galvanostat (Wuhan Corrtest Instruments Corp., Ltd., China) at room temperature (*ca.* 23 °C). The freshly prepared dispersions were loaded in a well-sealed Swagelok cell as described in ref. 4. The impedance spectra (at 100 mV) represented by Nyquist plots were fitted by an equivalent circuit to find out the resistivity of dispersions then the conductivity values were calculated.

An electrical equivalent circuit (inset of Fig. 6b) of the studied CB dispersions is proposed. It comprises an electronic R1 and an ionic R2 resistors, the latter in series with capacitive element Q1 of the double layer and Warburg element W3. The ionic branch accounts for the migration of ions and their accumulation at the surface of the electrodes, compensating their polarization, known as the double-layer. The electronic branch signifies the pure electronic resistance R1 due to the flow of electrons through the percolating CB network (pathways).^20^ This equivalent circuit has been edited in EC-Lab V10.20 (Bio-Logic Science Instruments, France), and used to fit the impedance spectra (Nyquist plots).

### 2.5. Optical and electron microscopy

Carbon black dispersions were observed by using Carl Zeiss (Axio Scope.A1) optical microscope, using polarized light source at different magnifications. The morphology and microstructure of CB and CNFs were examined by transmission electron microscope TEM (JEOL 100 CX). Trace amounts of CB and CNFs were dispersed in acetone in a bath sonication for few minutes then a drop of the prepared dispersion was dried on grid of copper before characterization. The scanning electron micrograph of CB-CNFs hybrid dispersion (Fig. 8b) was recorded by FEI Nova Nano SEM 450.

### 2.6. Raman spec

A ThermoFisher Scientific Raman spectrometer equipped with a microscope MPlan 10×/0.25 BD was used to obtain the Raman spectra of CB and CNFs after deposition of samples on a glass slide. Samples was excited with laser beam of 633 nm wavelength before spectra recording.

## 3. Results and discussion

### 3.1. Morphology and microstructure of carbon black (CB) and nanofibers (CNFs)

**Fig. 1:**
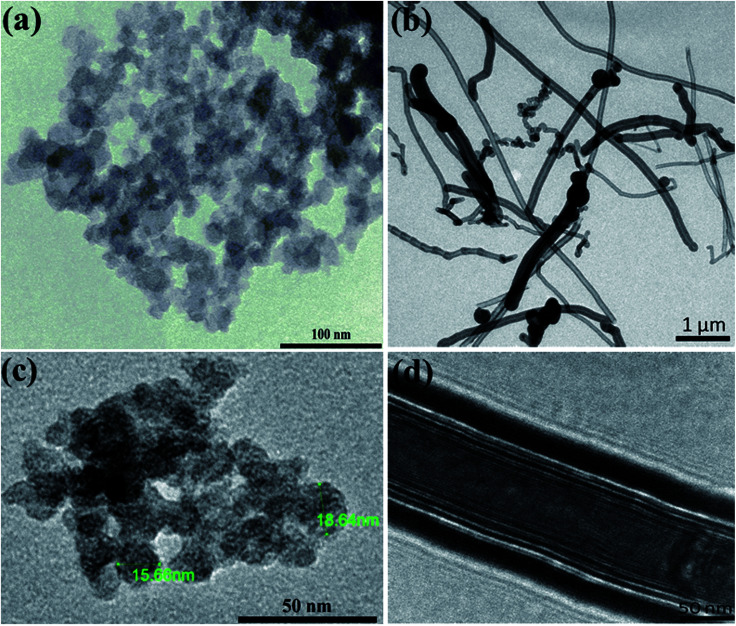
Transmission electron micrographs at different magnifications for (a and c) carbon black and (b and d) carbon nanofibers.

The carbon black (CB) is characterized by its semispherical nature of primary particles of an average size around 20 nm as can be seen from TEM micrographs (Fig. 1a). The primary particles are fused together to form branches which in turn construct the aggregates of carbons. The aggregates get closer to eventually form the agglomerates. This is the general microstructural picture of the CBs in the dry state as revealed either by TEM micrographs or even in aqueous dispersions as will be discussed below. Fig. 1b, on the other hand, demonstrates the filamentous morphology of carbon nanofibers (CNFs) which are composed of folded graphene sheets with hollow core along the nanofiber. The CNFs have high aspect ratio (length/diameter) with an average diameter of 130 nm and length ranging from 2 to 20 μm; typical dimensions of CNFs.^21^

High-resolution transmission electron micrographs reveal deeper insight on the microstructure and degree of graphitization of carbon nanomaterials. As can be seen from Fig. 1c, the TEM lattice fringe of CB shows spherules with characteristic concentric microtexture of turbostratic structure where the graphitic layers are rotationally misaligned showing some degree of graphitization.^22^ This is a typical microstructural picture of amorphous spherical carbon blacks.^23,24^ On the other hand, the TEM micrographs of CNFs (Fig. 1d) demonstrates the long and straight morphology of nanofibers with large hollow core (*ca.* 100 nm) along the fiber length. It is obvious the high degree of graphitization of the nanofibers since well-aligned graphite layers are stacked. This kind of nanofibers have a coated layer of amorphous carbon at the outer part of the nanofiber due to the secondary thickening growth process during CNFs manufacturing.^21^

**Fig. 2:**
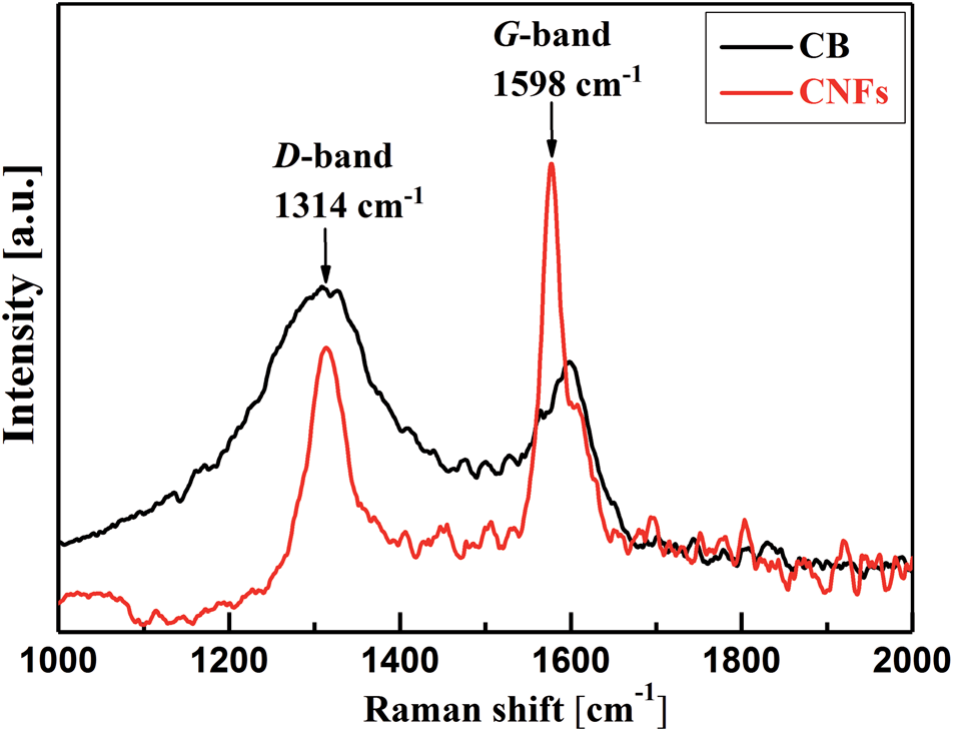
Raman spectra of carbon black (CB) and carbon nanofibers (CNFs).

Deeper insight into the microstructure of CB and CNFs is elucidated from the analysis of Raman spectra. As can be seen from Fig. 2, the two carbon nanomaterials show two characteristic bands at about 1314 cm^−1^ and 1598 cm^−1^. The former band is known as D-band (D denotes defects) which is due to the presence of defects (disordering) in the C–C lattice structure of the carbon nanomaterials. The second feature band at 1598 cm^−1^ is the G-band (G denotes graphitic) which is associated with the graphitized carbon structure where the C-atoms are sp^2^ hybridized.^25^ The relative intensities of the D- and G-bands (*I*_D_/*I*_G_) can be taken as indicator for the degree of graphitization of carbons. In comparison, CNFs show lower *I*_D_/*I*_G_ (∼0.54) than that of CB (∼1.4) implying the higher degree of graphitization of nanofibers than amorphous carbon black. Such highly graphitic structure is associated with higher electrical conductivity and therefore addition of CNFs to CB may improve the overall electrical conductivity of their hybrid (CB-CNFs) dispersions as will discussed below.

### 3.2. Effect of lithium salt on carbon black dispersion

**Fig. 3:**
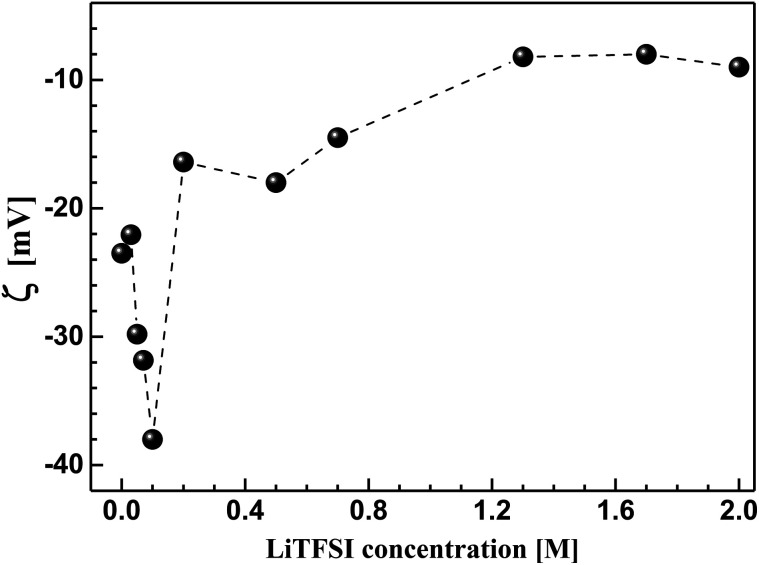
Effect of lithium bis(trifluromethanesulphonyl)imide (LiTFSI) concentration on the zeta potential (*ζ*) of carbon black in aqueous medium at 25 °C.

In order to understand the bulk rheological properties of CB dispersions in aqueous medium, it is important to investigate the effect of lithium bis(trifluromethanesulphonyl)-imide (LiTFSI) on the CB dispersions. This can be achieved by following the change of zeta potential (*ζ*) with the salt concentration as depicted in Fig. 3. The salt-free CB dispersion shows zeta potential value of *ca.* −25 mV. This indicates the negatively charged nature of the carbon surface as a consequence of possible dissociation of the surface's oxygen-functional groups (*e.g.* carboxylic group) in aqueous medium. On addition of lithium salt, the absolute value of zeta potential (|*ζ*|) strongly increases up to *ca.* 38 mV at 0.10 M LiTFSI. Such increase in |*ζ*| is likely attributed to the deprotonation oxygen-surface functional groups (such as carboxylic groups) rendering the CB particles more negatively charged.^26^ Above 0.10 M salt, |*ζ*| again decreases until nearly reach constant value of 10 mV at 1.0 M LiTFSI indicating the accumulation of the adsorbed Li^+^ ions on the carbon surface. It is worth mention that no charge reversal has been observed indicating that the CB particles remain negatively charged even at very high salt concentration (up to 2.0 M). Such decrease in zeta potential was previously reported for ketjen black in organic solution of LiTFSI and was attributed to the compression of the electrical double layer.^4,27^ Accordingly, the lithium salt is likely to induce a strong electrostatic screening effect resulting in a strong tendency of the CB particles to flocculate, in agreement with the fact that particles having *ζ* ≈ ±30 mV demonstrate significant agglomeration behaviour.^26^

In the current study, 1.0 M LiTFSI aqueous solution has been selected as a dispersing medium for carbon black in order to study the rheological and electrical properties of its dispersions. As a kind of comparison, this concentration is identical to the one used in previous studies of different carbon blacks (*e.g.* ketjen black)^4^ and carbon nanofibers^17^ in organic media (propylene carbonate).

### 3.3. Percolation of aqueous dispersions of carbon black – the rheological behavior

The structured CB dispersions are sensitive to various factors including the type and nature of CB particles,^19,28,29^ the nature of dispersing medium^30,31^ and the preparation method.^4,28,29^ In order to precisely determine the percolating threshold (C*), consistent formulation and measurement protocol were applied to all dispersions as described in the Experimental section (Sections 2.1 and 2.2).

#### 3.3.1. Dynamic strain sweep of CB dispersions

**Fig. 4:**
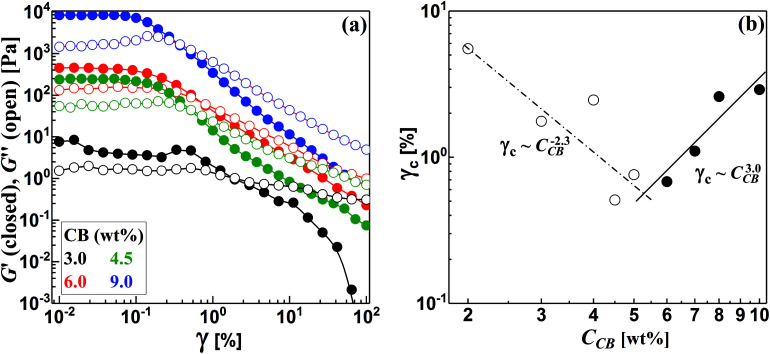
(a) Strain sweep test for some carbon black dispersions, and (b) variation of critical strain (*γ*_c_) with the concentration of carbon black (*C*_CB_).

Strain sweep test is a dynamic rheological experiment where the variation of dynamic viscoelastic moduli (*G*′; storage modulus and *G*′′ loss modulus) are monitored with the applied shear strain (*γ*) at constant frequency (1 Hz). This is a destructive test when the strain exceeds a critical value (*γ*_c_) below which the microstructure of dispersions is not perturbed; *i.e.* the linear viscoelastic regime where a minimal disturbance of the microstructure is applied. As can been seen from Fig. 4a, the CB dispersions, at low strain, show gel-like response where the storage moduli (*G*′) are higher than loss moduli (*G*′′) and both moduli are independent of the strain. Above specific *γ*_c_, the moduli show *G*′–*G*′′ crossover beyond which the dispersions are liquid-like where *G*′′ is higher than *G*′ and both moduli strongly decrease. This is certainly related to the strain-induced structural transitions when the network of carbon black breaks up under strain when it exceeds *γ*_c_. It is rational to note that as the concentration of CB increases, the dynamic moduli monotonically increase and the *G*′–*G*′′ crossover shifts to lower strain (Fig. 4a). However, the dispersions below 2.0 wt% (data not shown) exhibit liquid-like behavior with *G*′′ > *G*′ and both dynamic moduli are independent of the strain over the entire range implying that the diluted dispersions lack strain-induced structural transitions, *i.e.*, the dispersions are not yet structured.

It is worth noting that the CB dispersions exhibit a strain overshoot where the *G*′′ shows a slight increase at moderate strains before a sudden decrease and crossover of moduli where the dispersions are liquid-like (Fig. 4a). Previous research on suspensions of carbonaceous materials such as carbon black,^32^ nanotubes (CNTs)^33–35^ and nanofibers (CNFs)^17^ reported similar behavior which was ascribed to the formation of a temporary network able to resist the deformation but enough weak to breaks up and showing strain-weakening above the critical strain (*γ*_c_).^36^ In general, the CB dispersions show mild strain overshoot behavior in comparison to that exhibited by dispersions of CNFs.^17^ Moreover, the behavior becomes more pronounced as the CB concentration increases in contrast to the trend exhibited by CNFs.^17^ Such varied trend between CB and CNFs may ascribe to the different nature of microstructures where the network of CB dispersions is composed of aggregates that are linked by rigid branches, which are easily broken under deformation. On the other hand, the network of CNFs dispersion is built of bundles linked by flexible nanofibers able to sustain the shear deformation.^17^

Interestingly, the variation of critical strain (*γ*_c_) with the concentration of CB shows two different regimes with different power-law exponents around 5.5 wt% CB as depicted in Fig. 4b. In the dilute regime (<5.5 wt% CB), *γ*_c_ monotonically decreases with the concentration of CB showing a scaling law: *γ*_c_ ∼ *C*_CB_^−2.3^ with negative exponent higher than that exhibited by fibrous carbons.^17,37^ This is likely attributed to the breaking up of the rigid branches between the aggregates of CB. Above 5.5 wt%, presumable saturated network of CBs is formed where dense strongly bonded aggregates require higher deformation to exhibit strain weakening so that *γ*_c_ of the concentrated dispersions show a positive scaling exponent (3.0) implying the stiffness of the saturated networks. Chakraborty *et al.* reported a similar increasing of *γ*_c_ with the polymer concentration in hydrogel system and attributed this trend to the increased density of crosslinking of polymer chains.^38^

#### 3.3.2. Dynamic frequency sweep of CB dispersions

**Fig. 5:**
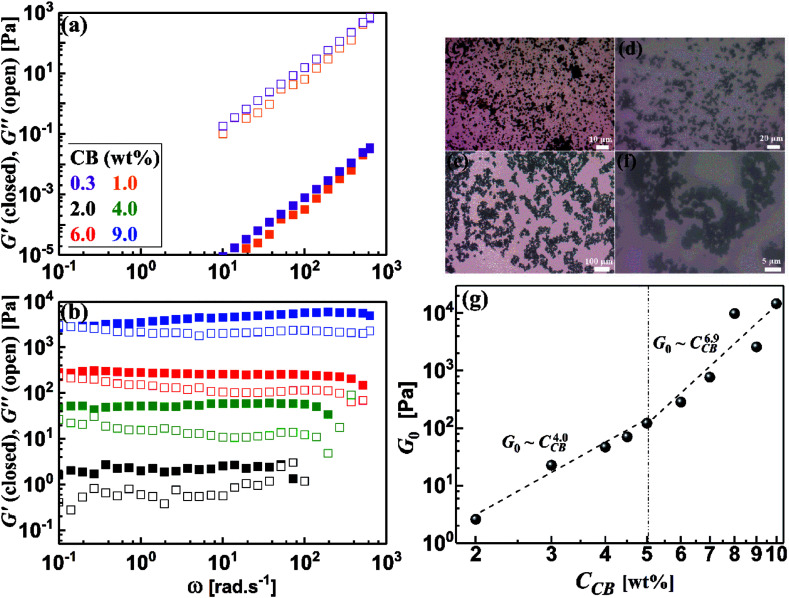
(a and b) Dynamic frequency sweep of carbon black dispersions, (c–f) optical micrographs of some CB dispersions, and (g) variation of plateau modulus (*G*_0_) with the concentration of carbon black (*C*_CB_).

The dynamic frequency sweep test is nondestructive dynamic rheology where a minimal strain is applied, in the linear viscoelastic regime, to induce flow without serious perturbation of the microstructure of dispersions. Hence, the structural evolutions and construction of the network in CB dispersions under equilibrium conditions can be examined by the frequency sweep measurements. Fig. 5a and b present the rheograms of dynamic frequency sweep for CB dispersions over wide concentration range. At low CB concentration (Fig. 5a), the dispersions show a liquid-like behavior where *G*′′ is higher than *G*′ and both moduli are dependent of the angular frequency (*ω*) indicating that the diluted dispersions are not yet structured. At *C*_CB_ ≥ 2.0 wt%, the rheological behavior thoroughly changes and the dispersions exhibit gel-like response where *G*′ is higher than *G*′′ and both moduli are nearly independent of the angular frequency (Fig. 5b) implying the onset of construction of CB network at 2.0 wt%. This behavior has been previously reported for analogous systems of carbon nanomaterials.^4,17,39^ The optical micrographs (Fig. 5c–f) confirm the evolution of CB microstructure as the concentration increases. The diluted dispersions have small aggregates (Fig. 5c), which slightly grow and form branches (Fig. 5d) as the CB concentration increases. When the CB concentration reaches the critical threshold, large agglomerates are formed and a three-dimensional “percolated” network of CB is constructed (Fig. 5e and f). The visual inspection of the evolution of microstructure of CB dispersion is in consistent with the development of rheological behavior as the CB content increases under equilibrium conditions.

It is rational to note that the magnitudes of the dynamic moduli increase with the CB concentration as can be seen from Fig. 5b. This behavior can be accurately followed up through the quantitative variation of plateau moduli (*G*_0_; which is defined as *G*′ when *ω* tends to zero) of dispersions with CB concentration as depicted in Fig. 5g. It should be stated that the diluted dispersions (*C*_CB_ < 2.0 wt%) have no *G*_0_ due to their liquid-like rheological behavior. Therefore, Fig. 5g starts at 2.0 wt% which is considered as the rheological percolating threshold. As the CB concentration increases, *G*_0_ monotonically increases showing a power law: *G*_0_ ∼ *C*_CB_^4.0^, with a scaling exponent of 4.0, which falls in the range of 3.7–4.5 reported for structured colloids.^40–43^ Previous studies on CB dispersions in propylene carbonate^17^ and silicon oil^39^ showed a similar exponent. Above 5.0 wt% CB, *G*_0_ shows higher scaling exponent of 6.9 which is similar to the one reported for nonaqueous dispersions of carbon nanofibers^17^ which may implies higher fractal dimensions.^44^

Development of the microstructure of CB network can be viewed through following the change in the fractal dimensions of the percolated network with the CB concentration. Considering the CB network is built up of uniformly packed isolated aggregates,^45^ the fractal dimension, expressed by packing index (*D*), is related to the exponent of the scaling law: *G*_0_ ∼ *C*_CB_^*n*^ as *D* = 3 − (2/*n*).^46^ The dilute CB dispersions (*C*_CB_ < 5.0 wt%) have a packing index *D* = 2.5 indicating a rapid diffusion-limited aggregation mechanism where the attractive forces between the aggregates results in an open fractal structure.^47^ Analogous dispersions of carbon blacks (ketjen black and Super C45) in organic medium showed identical packing index,^4^ and slightly lower index (2.24) was reported for dispersion of CB in silicon oil.^39^ In the concentrated regime (*C*_CB_ > 5.0 wt%), saturated network shows higher packing index (*D* = 2.7) implying more compact network dominated by repulsive interaction in agreement with Nakagawa model^48^ and densely-packed percolated carbon nanofibers in organic medium (1 M LiTFSI in propylene carbonate).^17^

It is well stated that the rheological behavior of CB dispersions is strongly dependent of the characteristics of the carbon black (surface area, surface chemistry, particle size and shape, *etc.*)^19^ and the nature of dispersing medium (wetting strength, pH, viscosity, *etc.*).^30,31^Table 1 depicts a comparison between the currently studied carbon black and previously studied ones^4^ in terms of the characteristics of carbons (particle size and surface area) and nature of dispersing medium. It is worth noting that the rheological percolating threshold of dispersions in organic medium (1 M LiTFSI in propylene carbonate) decreases as the particle size decreases (*i.e.* as the surface area increases). However, the dispersions in aqueous medium (1 M LiTFSI in H_2_O) show higher percolating threshold although the carbon black (PBX51) has much smaller particle size (higher surface area). This certainly ascribe to the nature of dispersing medium and its compatibility (wettability) with the carbon black so that hydrophobic carbon black is favorably wetted by organic medium than aqueous one. Hence, the currently studied carbon black (PBX51) shows higher percolating threshold than other carbon blacks (KB and C45). Moreover, the packing efficiency (expressed by the aspect ratio of primary particles) may play a role since the primary particles with higher packing efficiency exhibits higher percolating threshold.^49^

**Table 1:** Comparison between the characteristics of different types of carbon blacks (KB-EC300 and Super C45 from ref. 4 and the currently studied PBX51)

	PBX51	KB-EC300	Super C45
Particle size (nm)	20	300	725
BET surface area (m^2^ g^−1^)	1420	800	45
Rheological percolating threshold (wt%)	2	0.3	0.5
Dispersing medium	1 M LiTFSI in H_2_O	1 M LiTFSI in PC	

### 3.4. Percolation of aqueous dispersions of carbon black – the electrical behavior

**Fig. 6:**
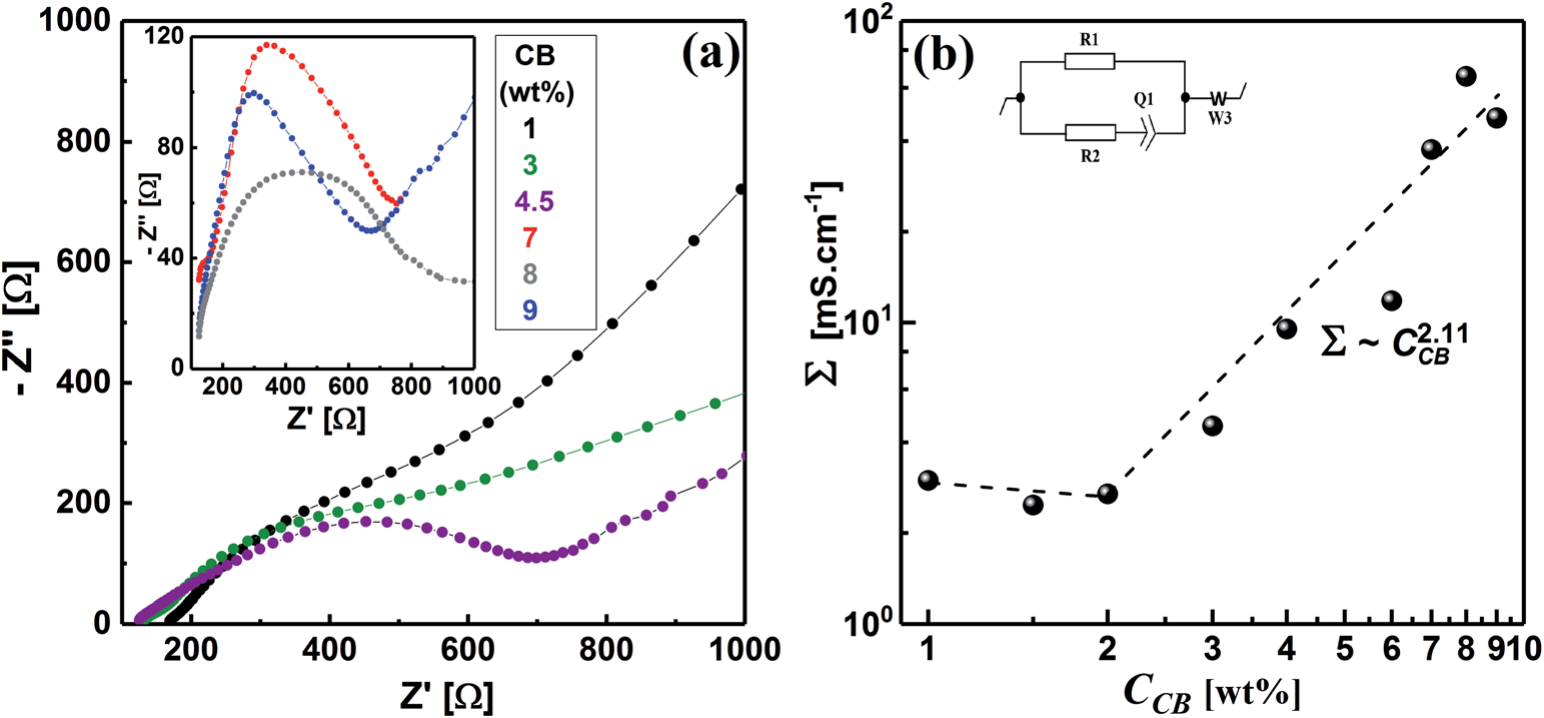
(a) Impedance spectra represented by Nyquist plots (imaginary part of impedance *Z′′ versus* the real part *Z*′), and (b) variation of the electrical conductivity (*Σ*) with the carbon black concentration (*C*_CB_), for the CB dispersions at ambient temperature (*ca.* 23 °C). The inset of (b) is the equivalent circuit used to model the behavior of the dispersions: it is composed of an electronic R1 and an ionic R2 resistors, the latter in series with capacitive element Q1 of the double layer and Warburg element W3. EC-Labs software (Bio-Logic Science-Instruments) has been used to fit the Nyquist plots.

Electrochemical impedance spectroscopy (EIS) is a nondestructive technique, which has been extensively used to elucidate the microstructure of carbon dispersions.^4,17,50–52^ The evolution of the microstructure in CB dispersions can be inspected by observing the development of the impedance spectra, represented by Nyquist plots, with the CB concentration. Fig. 6a demonstrates the Nyquist plots in the form of variation of imaginary part of impedance (*Z*′′) with the real analogy (*Z*′) for some CB dispersions over wide CB concentration range. It can be seen that the diluted CB dispersions (1 wt%) show nearly linear Nyquist plot similar to that of the dispersing medium (1 M LiTFSI in H_2_O) implying the ionic nature of the dispersions. As the CB concentration increases, the Nyquist plot is dominated by a typical semicircle (in the high-frequency regime) and linear tail (in the low-frequency regime) implying a mixed kinetic and charge-transfer mechanisms. Similar Nyquist plots have been shown by graphite- and graphite-based electrode materials.^53,54^ In the high-frequency regime, the semicircles become narrower as the CB concentration increases indicating the construction of conductive network in CB. On the other hand, the slope of line at low frequency remains nearly constant at ∼0.5 and increases to ∼1 at very high CB concentration implying the development of CB network towards more compactness and hence restriction of lithium-ions diffusion in the saturated network at very high CB content.

Upon application of a potential difference between the two metallic electrodes of the Swagelok cell, the current response is due to the diffusion of ions and the electronic flow in the percolated network.^55^ In order to account for the development of CB network, the electrical conductivity of dispersions has been quantitatively extracted from fitting the Nyquist plots with an equivalent circuit (inset of Fig, 6b). This is can be achieved by calculating the electrical conductivities (*Σ*) – considering the geometry of cell – of dispersions from the pure electronic resistance R1 which takes into account the circulation of the electrons through the percolating network of CB. As can be seen from Fig. 6b, the electrical conductivity is nearly independent of the CB content in the dilute regime implying that the CB network has not yet fully constructed and less-dense effective chains are fairly in direct contact to transmit an electrical current.^4,56^ Above 2.0 wt% CB, the conductivity steeply increases exhibiting a power-law relation: *Σ* ∼ *C*^2.11^, with an exponent close to the theoretically calculated one for three-dimensional network at the percolating threshold,^57,58^ and the reported exponents for analogous system of carbon blacks: Super C45 in organic medium^4^ and acetylene black in copolymer solution.^59^ It is rational to expect that CB with smaller particle size would form highly dense network, however, the wettability of solvent again dominates to control the network morphology so that the currently studied CB forms less-dense network than that formed by ketjen black (KB) and almost similar to that of Super C45 in organic media.^4^ Moreover, the aqueous dispersions of the currently studied CB have 100-fold electrical conductivity higher than those of KB and Super C45 in organic medium over the same concentration range. This is likely attributed to the more numerous conducting paths due to smaller particle sizes resulting in a higher density of effective chains. In addition, the highly graphitic structure of the currently studied CB with less surface functional groups as indicated from the zeta-potential value of salt-free dispersion (Fig. 3).

Fig. 6b shows a critical CB concentration of 2.0 wt% above which the electrical conductivity of dispersions abruptly increases. This critical concentration is the “electrical” percolating threshold at which the density of the effective chain increases and 3D network is constructed. Interestingly, both the rheological and electrical percolating thresholds of the currently studied CB in aqueous medium coincide. Dispersions of carbonaceous materials in organic media showed similar trend.^4,60,61^ When the conductive paths are formed across the material from one end to the other, the network is electrically percolated^62^ enough to transmit an electrical current in tandem with the formation of enough dense chains to resist the flow, *viz*., rheologically percolated.

To recap, the aqueous dispersions of the currently studied CB (with its high surface area and small particle size) show relatively high percolating threshold at 2.0 wt%, separating liquid-like rheological behavior at low concentration and gel-like behavior above 2.0 wt% where 3D network is constructed when the dense chains are fully extended through the dispersions. In comparison to analogous dispersions of carbon blacks in organic media,^4^ the aqueous dispersions of the currently studied CB show relatively high percolating threshold (2.0 wt%), mainly due to the less wettability of hydrophobic CB particles by polar medium. However, they show lower rheological dynamic moduli and much higher electrical conductivity over a similar CB concentration range to the previous study.^4^ These findings suggest that the aqueous formulation of this type of CB is an excellent conductive additives for electrodes suspensions of semi-solid redox flow cells.

### 3.5. Shear-induced transitions in CB dispersions

As described above, the non- destructive dynamic rheology (frequency sweep) could differentiate between two behaviors around the percolating threshold (2.0 wt%); liquid-like and gel-like behaviors. Now, it is vital to study the effect of shear flow on the microstructure of CB dispersions and elucidate any possible shear-induced structural transitions due to flow. This can be done through the steady-statue rheology which is a destructive test through which we can follow the variation of steady-shear viscosity (*η*) with shear rate (*γ̇*).

**Fig. 7:**
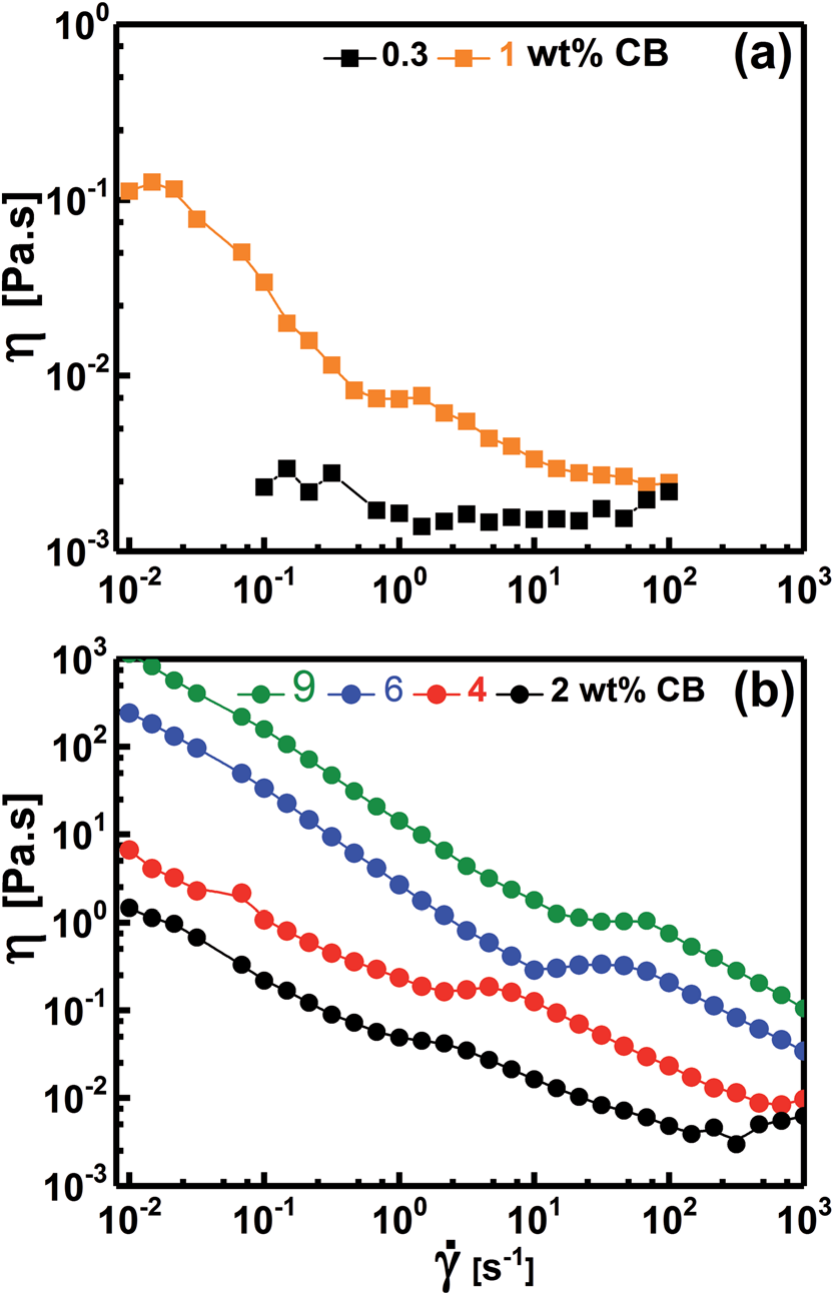
Variation of steady-shear viscosity (*η*) with shear rate *γ̇* for selected CB aqueous dispersions.

Fig. 7 demonstrates the dependence of the viscosity on the shear rate for diluted (liquid-like; Fig. 7a) and concentrated (gel-like; Fig. 7b) dispersions. At low CB concentration (*e.g.* 0.30 wt%, Fig. 7a), the dispersions show Newtonian behavior where *η* is independent of *γ̇* implying that the CB network has not yet constructed in agreement with the dynamic behavior where *G*′′ was higher than *G*′ (Fig. 5a), *i.e.*, liquid-like behavior. When the dispersions become percolated (*C*_CB_ ≥ 2.0 wt%), they exhibit a complex non-Newtonian behavior where *η* is strongly dependent on *γ̇* as shown in Fig. 7b. The dispersions show a three-regime flow curve: two shear-thinning (decrease in *η*) regions separated by a shear-thickening (increase in *η*) region. This behavior has been previously reported for analogous systems of carbon dispersions.^4,13,17^ This complex behavior is related to shear-induced microstructure transitions and can be explained as follow: (i) the shear-thinning at low *γ̇* is ascribed to breaking up of the percolated network into relatively small agglomerates, (ii) such agglomerates are likely to be enough anisometric (has aspect ratio) to erode and show shear-thickening (instability region) at a critical shear rate which shifts to higher rates as the CB concentration increases, and (iii) these anisometric agglomerates are seemed to align as *γ̇* increases showing the second shear-thinning region. Such complex non-Newtonian rheological behavior is in accordance with the gel-like nature (Fig. 5b) of percolated dispersions. However, this is not the case with the CB dispersion at 1.0 wt% which exhibits similar non-Newtonian behavior at low *γ̇* (Fig. 7a) although it showed liquid-like response (Fig. 5a). It is worth to note the presence of Newtonian region at low *γ̇* before exhibiting the complex three-regime flow curve as *γ̇* increases. This unusual behavior may ascribe to the presence of agglomerates which do not reach yet the percolation state so that it is liquid-like (Fig. 5a) and Newtonian (Fig. 7a) under equilibrium conditions. When the structure is perturbed at higher *γ̇*, the agglomerates are likely to suffer breaking up (first shear-thinning), erosion (shear-thickening) and alignment (second shear-thinning).

In conclusion, the aqueous dispersions of CB are Newtonian fluids below the percolating threshold and turn into non-Newtonian structured fluids above the threshold exhibiting a complex behavior (two shear-thinning regions separated by shear-thickening at intermediate rates). This complex behavior has been previously reported in analogous organic-based dispersions,^4^ however, the currently studied dispersions show lower magnitudes of viscosity over the same CB concentration range. Such low viscosity suggests that the aqueous dispersions of this CB are an excellent candidate to be conductive additives for anolytes and catholytes of semi-solid redox flow cells.

### 3.6. Hybrid carbon nanomaterials

Conductive additives are very crucial component of electrode slurries for energy storage systems such as solid-state ion-batteries and semi-solid redox flow batteries. Specifically, the semi-solid redox flow battery requires suspension electrodes (anolyte and catholyte) composed of high content of electroactive materials (electrically insulating) homogenously dispersed in the fully percolated network of carbon additives in an electrolyte (aqueous or organic medium). There is a maximum capacity of loading of electroactive materials above which the percolated network of CB is ruptured and the electrode suspension losses its electrical conductivity.^4,13,17^ In addition, the CB dispersions showed strong shear-induced structural transition and then varied electrical conductivity under flow.^29^ This transition was attributed to breaking up of the CB branches and then serious loss of conductivity.^4^ The dispersions of another allotrope of carbonaceous materials; the so-called carbon nanofibers (CNFs), nearly showed similar rheological behavior but was attributed to shear-induced alignments of fibers.^17^ This interesting difference in shear-induced structural evolution inspired Youssry and coauthors to mix CBs with CNFs to form hybrid carbon dispersions: (i) able to sustain higher content of electroactive materials without serious effect on the percolated network, and (ii) to overcome or compensate the conductivity loss due to breaking up of CB branches under flow condition.^17^

**Fig. 8:**
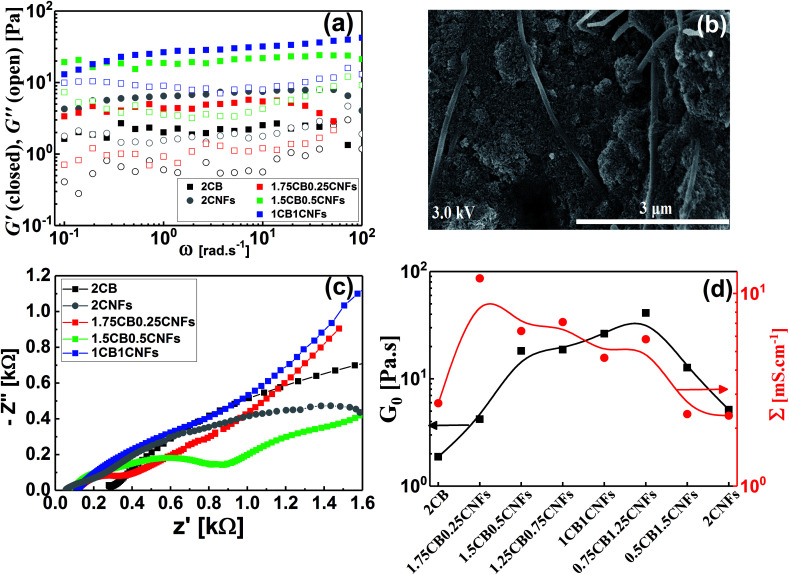
(a) Dynamic frequency sweep, (b) scanning electron micrograph, (c) impedance spectra (Nyquist plots), and (d) variation of plateau modulus (*G*_0_) and electrical conductivity (*Σ*) for hybrid carbon materials. The concentration of carbon materials is represented by wt% (*e.g.* 2CB, and 1.75KB0.25CNFs denote, respectively, 2.0 wt% CB, and 1.75 wt% CB + 0.25 wt% CNFs, *etc.*).

Analogously, we attempt to formulate and understand the rheological and electrical properties of aqueous dispersions of mixed CB and CNFs (hybrid carbons) merging the advantages of both carbonaceous materials. At its percolating threshold (2.0 wt%), amounts of CB was replaced by trace equivalent amounts of CNFs, keeping the overall carbon content at 2.0 wt%. Fig. 8 shows the dynamic rheological and electrical properties of dispersions of hybrid carbons (CB-CNFs), and their relation with the equilibrium microstructure at no perturbation.

The dynamic viscoelastic properties of CB, CNFs and their hybrid dispersions are depicted in Fig. 8a. The aqueous dispersions of pure CB and CNFs at 2.0 wt% exhibit gel-like viscoelastic behavior where the storage modulus (*G*′) is higher than the loss modulus (*G*′′) and both moduli are nearly independent of the angular frequency (*ω*), and the magnitudes of dynamic moduli of CNFs dispersion are slightly higher than those of CB dispersion. This behavior is consistent with varied particle morphology and hence the aggregation mechanisms between isometric spherical particles of CB and anisometric fibers of CNFs. The aggregate network of CNFs (assuming it is percolated) is composed of large aggregates (flocs) and connecting bundles of nanofibers which show higher rigidity than that of smaller aggregates linked by relatively weak CB branches.^17^ Upon replacing an amount of CB by an equivalent amount of CNFs, the dispersions still behave gel-like viscoelastic behavior but the magnitude of dynamic moduli is increased until a critical content of CNFs beyond which the magnitudes of the moduli decrease. The increased rigidity can be explained in the light of formation of a cooperatively supporting network^63,64^ of randomly distributed CNFs and CB particulates where the CNFs, at low content, act as long-distance wires and the particulate CB forms local conductive paths acting as interconnections between the nanofibers. SEM micrograph (Fig. 8b) supports this explanation where the CNFs are likely to be embedded in the CB matrix. As the CNFs content increases, the CNFs are bundled so that it breaks up the CB branches and then the rigidity of the hybrid dispersions diminishes. Fig. 8d quantitatively demonstrates the variation of rheological plateau modulus (*G*_0_) with the CNFs content. As can be seen, *G*_0_ exhibits inverted bell-like curve showing the existence of a critical CNFs content (0.75–1.0 wt% CNFs) beyond which the increased content of CNFs seriously breaks up the 3D network of hydride dispersions resulting in losing the cooperative supporting. Similar behavior has been reported previously for analogous hybrid carbon dispersions in organic medium^17^

The impedance spectra of some hybrid dispersions, represented by Nyquist plots, are demonstrated in Fig. 8c. In general, the hybrid dispersions show complex Nyquist plots composed of two fused semicircles at low frequency and tail at high frequency, depending on the content of CNFs. An equivalent circuit composed of three resistors and two capacitive elements has been used to fit the impedance data, where the resistance values at low frequency have been considered to calculate the electrical conductivities of dispersions. As shown in Fig. 8d, the electrical conductivity of hybrid dispersions shows inverted bell-like curve, in agreement with the behavior of rheological plateau modulus. Small amount of CNFs (0.25 wt%) strongly increases the conductivity of its hybrid dispersion before the conductivity gradually decreases as the CNFs concentration increases. This trend can be explained on the basis of formation of cooperatively supporting network at small CNFs where the fibers are randomly distributed in the dispersions and wiring the CB particulates, therefore, the conductivity increases. At higher concentration of CNFs, the formation of CNFs bundles is likely to be responsible to the potential loss of electronic writing and hence a drop in conductivity of dispersions due to rupturing of branches between CB particulates. It is well known that bundled state of CNFs suffer a significant loss of conductivity because of the electric current only flows on the outermost fibers in bundled CNFs, while the inner fibers do not contribute significantly to the current.^17,65^ Therefore, increasing of the CNFs content beyond the critical value has negative effect on the electrical conductivity of hybrid dispersions.

**Fig. 9:**
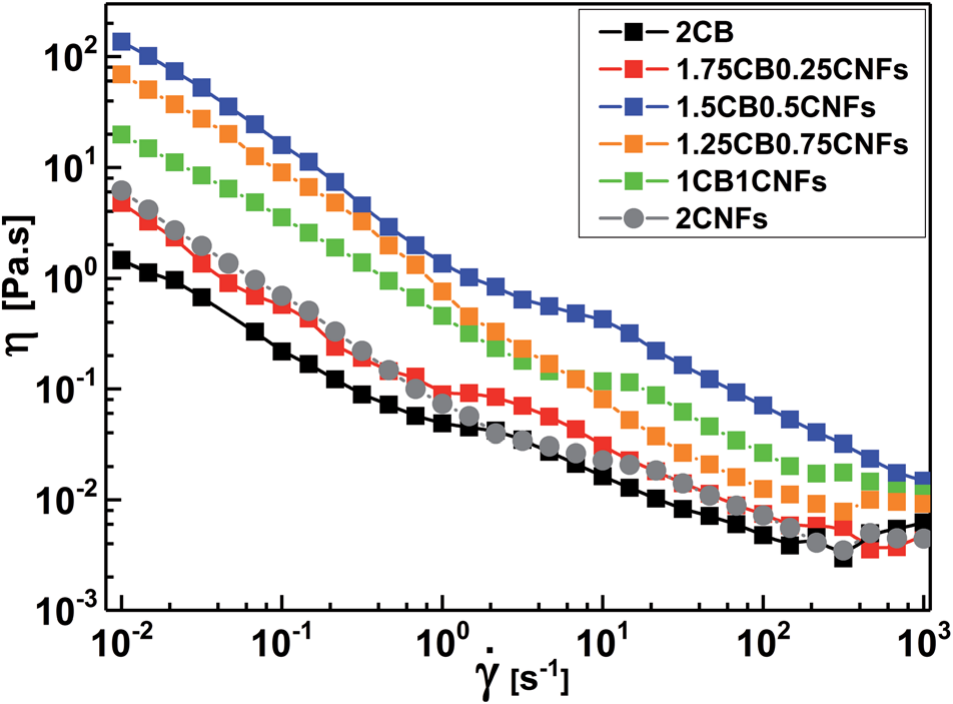
Variation of steady-shear viscosity (*η*) with shear rate *γ̇* for aqueous dispersions of 2.0 wt% CB (2CB), 2.0 wt% CNFs (2CNFs), and some selected hybrid dispersions (*e.g.* 1.75KB0.25CNFs denotes 1.75 wt% CB + 0.25 wt% CNFs).

Now, it is interesting to account for the possible shear-induced structural/alignment transitions of the hybrid dispersions under continuous shear flow as simulating conditions for behavior of electrode suspensions in semi-solid redox flow cells. As can be seen from Fig. 9, the hybrid dispersions exhibit the three-regime non-Newtonian behavior, which commonly exhibited by the CB and CNFs aqueous dispersions and previously reported for analogous systems.^4,13,17,66^ As can be seen from Fig. 9, the three-regime flow curve is dominated by two shear-thinning regimes at low and high shear rates separated by shear-thickening region at intermediate rates. This behavior indicates a shear-induced structural transition in the dispersions under flow. It is worth mentioning that the particle morphology of CB (isometric particles) is different from that of CNFs (anisometric fibers) and hence different aggregation mechanisms emerge.^17^ At constant concentration (2.0 wt%), the CNFs dispersion (with aggregated network of bundles linked with flexible fibers) shows higher plateau modulus (rigidity) than that of analogous CB dispersion (with aggregated network of flocs linked by CB branches) (Fig. 8a and d). Consequently, the CNFs dispersion rationally shows higher viscosity than its analogous CB dispersion at the percolating threshold. Accordingly, the initial shear-thinning, at low shear rates, originates from the breaking up of the large aggregates into smaller flocs. These flocs are enough anisometric to erode and the dispersions exhibit shear-thickening over moderate shear rates. The thickening occurs at a critical shear rate which is higher for CNFs dispersion than CB one implying the strength of CNFs network. The second shear-thinning region, at high shear rates, originates from the alignment of anisometric flocs in the flow direction.

At the first glance, the hybrid dispersions generally show the three-regime flow curve indicating the shear-induced structural transitions under shear flow (Fig. 9). As the CNFs content increases, the viscosity increases until 0.50 wt% CNFs beyond which the viscosity again decreases. This trend is nearly consistent with the behavior of hybrid dispersions under quiescent state (Fig. 8a) where the small amounts of CNFs (0.25–0.50 wt%) are likely to reinforce the CB network by wiring the CB aggregates^13^ when the CNFs are in the flexible nonaggregated state (below its percolating threshold). When the CNFs content exceeds its percolating threshold (>0.50 wt%), the aggregated “bundled” nanofibers exhibit a synergetic effect with the CB aggregates which are sensitive to shear flow so that the viscosity decreases. This behavior has been previously reported for analogous dispersions.^17^ It is also worth noting that the shear-thickening region is monotonically shifted to higher shear rates on increasing the CNFs content implying the cooperative supporting of the CB network by the addition of CNFs.

In summary, the hybrid dispersions of spherical carbon black and fibrous carbon nanofibers in aqueous medium offer enhanced rheological and electrical properties merging the advantages of both types of carbon materials when an optimal content nanofiber was selected which certainly depends on the surface chemistry and particle size of carbon materials as well as the wettability of solvent. This hybrid dispersion offers a conductive 3D network able to sustain higher load of electrical insulating electroactive materials and resist the serious decrease in conductivity (due to rupturing of conductive pathways) under flow conditions *via* writing the ruptured aggregates by filamentous nanofibers. Accordingly, the hybrid dispersions of CB and CNFs are excellent conductive additive for electrode suspensions in semi-solid redox flow batteries.

## 4. Conclusions

In this study, we systematically demonstrated the structural evolution and shear-induced transition exhibited by dispersions of high-surface area carbon black (CB) and its hybrid dispersions with carbon nanofibers (CNFs) in an aqueous electrolyte (1 M lithium bis(trifluoromethanesulfonimide) in water) at ambient temperature. The combination of rheology and impedance spectroscopy proved to be powerful techniques to simultaneously elucidate the development of microstructure and precisely determine the percolating threshold in the aqueous dispersions of CB as well as sensitively suggest the optimal composition of CB-CNFs hybrid dispersion as a plausible conductive dispersion for aqueous semi-solid redox flow cells.

In aqueous electrolyte, the currently studied CB exhibited a structural transition from liquid-like to viscoelastic gel-like behavior in tandem with the change of the shape of Nyquist plot from linear to semi-circle at a high percolating threshold of 2.0 wt%. Interestingly, the rheological and electrical percolating thresholds coincide, implying the unusual development of 3D open network upon increasing the CB content, which is mainly controlled by the affinity of the aqueous medium towards CB particles rather than its small particle size. In spite of its high percolating threshold, the aqueous dispersions of CB showed lower plateau modulus and much higher electrical conductivity in comparison to analogous dispersions in organic medium. Moreover, the CB dispersions exhibited shear-induced structural transitions represented by complex three-regime non-Newtonian flow curve with two shear-thinning regions at low (due to breaking up of large aggregates) and high (due to alignments of flocs) shear rates separated by shear-thickening region (due to erosion of anisometric flocs) over intermediate shear rates.

At the percolating threshold of CB (2.0 wt%), replacing a trace amount of CB by an equivalent amount of carbon nanofibers (CNFs; optimally between 0.25 and 0.50 wt%) resulted in hybrid dispersions with 3D network dominated by cooperative supporting from the fibrous flexible nanofibers which enhanced the electrical conductivity at rest and possibly offered a recoverable conductivity under shear flow. These hybrid dispersions are promising alternative to the pure CB ones as an electrically conductive matrix in aqueous semi-solid redox flow cells, which is expected to sustain higher load of electrically insulating electroactive materials without significant loss of conductivity either at rest or under flow conditions. Work is in progress to examine the electro-rheological and electrochemical performance of a real anolyte and catholyte based on hybrid dispersion in aqueous medium.

## Conflicts of interest

There are no conflicts to declare.

## Supplementary Material
